# Integrating single-cell sequencing and clinical insights to explore malignant transformation in odontogenic keratocyst

**DOI:** 10.1016/j.csbj.2025.03.027

**Published:** 2025-03-18

**Authors:** Guile Zhao, Yike Li, Hongling Li, Mingzhe Bao, Grace Paka Lubamba, Guanru Wang, Bo Han, Yaling Tang, Taiwen Li, Chunjie Li

**Affiliations:** a State Key Laboratory of Oral Diseases & National Center for Stomatology & National Clinical Research Center for Oral Diseases, Chengdu, Sichuan 610041, China; b Department of Oral Pathology, West China Hospital of Stomatology, Sichuan University, Chengdu 610041, China; c Department of Head and Neck Oncology, West China Hospital of Stomatology, Sichuan University, Chengdu 610041, China; d Department of Oral and Maxillofacial Surgery, University Clinics of Kinshasa, Faculty of Dental Medicine, University of Kinshasa, Kinshasa B.P.127, Congo

**Keywords:** ScRNA-seq, Odontogenic keratocyst, Malignant transformation, Myofibroblast, Neuroinvasive, Lymph node metastasis

## Abstract

The malignant transformation of odontogenic keratocysts (OKC) into cancerous odontogenic keratocysts (COKC) is exceedingly rare, and its mechanisms remain poorly understood. Studies exploring the cellular heterogeneity, molecular pathways, and clinical features of COKC are limited. In this study, we performed single-cell RNA sequencing (scRNA-seq) on three COKC samples and integrated the data with a public OKC dataset, identifying 22,509 single cells. Two COKC-specific epithelial subpopulations, Basal-C0-EXT1 and Basal-C3-HIST1H3B, were identified. These subpopulations exhibited enhanced stemness and invasive potential, respectively, suggesting their roles as key drivers of OKC carcinogenesis. Fibroblasts underwent phenotypic transitions, particularly from inflammation-associated fibroblasts (IFBs) to myofibroblasts (MFBs). Similarly, macrophage phenotypic transformation may also play a role in OKC carcinogenesis. Clinical observations of severe lesion-area pain in COKC patients suggest potential neuroinvasiveness, Supported by single-cell transcriptomic data, imaging findings, and histopathological evidence. A review of clinical data revealed that none of the COKC patients exhibited cervical lymph node metastasis. Single-cell transcriptomics suggests that this phenomenon may be associated with an active immune microenvironment in COKC, reduced epithelial-mesenchymal transition (EMT) activity, lower VEGFC expression, and upregulated MAST4 expression as a potential regulator of lymphatic metastasis. In conclusion, COKC exhibits distinct molecular, cellular, and clinical characteristics compared to OKC, featuring potent neuroinvasiveness and low lymph node metastatic potential. These findings provide important insights into the mechanisms underlying COKC development and may guide novel diagnostic and therapeutic strategies.

## Graphical Abstract

**Figure:**
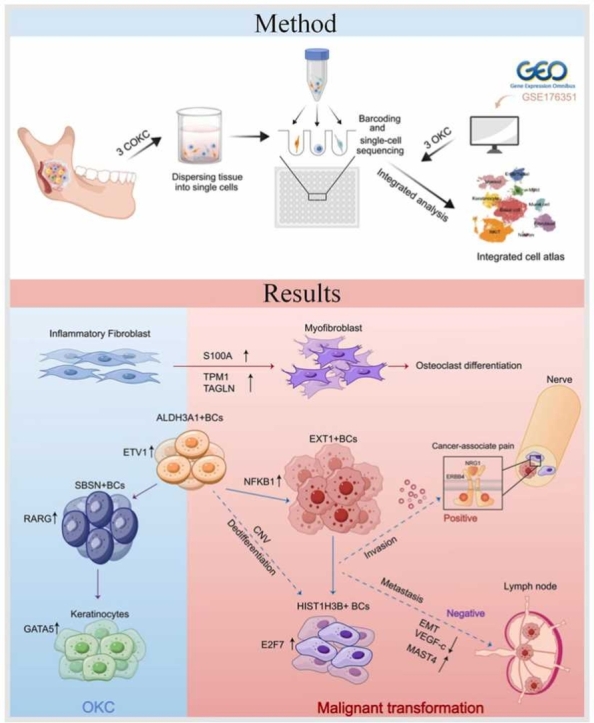

## 1 Introduction

Odontogenic keratocyst (OKC) is a common odontogenic cyst, accounting for approximately 10 % of all such cystic entities [1]. Unlike other types of odontogenic cysts, OKC tends to exhibit an infiltrative growth pattern and aggressive biological behavior [2], [3], [4], [5]. Although the potential for malignant transformation into primary intraosseous carcinomas (PICs) exists, such cases are exceedingly rare in clinical practice, with an incidence rate of approximately 0.55 % [6]. In this article, we refer to PICs originating from the primary lesions of OKCs as cancerous odontogenic keratocyst (COKC). Research on COKC is limited, primarily based on isolated case reports. When examined under optical microscopy, areas of malignant transformation within OKC generally retain key pathological features of OKC, such as a uniform epithelial cell layer thickness, enhanced staining of basal cells, and expansion of polymorphic cells on the surface [7]. Some case studies have highlighted the utility of immunohistochemical examination, particularly the evaluation of p53 and Ki67 markers, in distinguishing between OKC and its resultant PICs [6], [8].

In the therapeutic management of COKC, surgery stands out as the preferred approach, including tumor excision, ipsilateral neck lymph node dissection, and reconstructive procedures [6], [9]. However, due to the aggressive nature of COKC, local recurrence emerges as a primary cause of treatment failure. Studies have suggested the possibility of COKC recurrence within a year, highlighting that relying solely on surgical interventions may not be sufficient for complete tumor eradication [9]. Hence, developing a precise therapeutic strategy that effectively eliminates the tumor while minimizing damage to surrounding normal structures is crucial. A comprehensive understanding of the biological characteristics and underlying molecular mechanisms of COKC is essential to achieving this goal.

Research progress on OKC and its malignant transformation is hindered by the lack of key models, such as cell lines and in vivo tumorigenesis models [10]. Previous studies have mainly focused on histopathology and limited molecular markers, leaving gaps in the understanding COKC's cellular and functional aspects [8], [11]. Single-cell RNA sequencing (scRNA-seq) has revolutionized the exploration of tumor development mechanisms at the molecular level, enabling the tracking of developmental pathways of different tumor cell subtypes and providing insights into tumor heterogeneity and the complexity of the tumor microenvironment (TME) at the single-cell level [12], [13], [14]. In this study, we utilized scRNA-seq to analyze three rare COKC samples collected by our team and integrated these data with those from three additional non-malignant OKC samples obtained from public databases. This approach aimed to elucidate the functional characteristics of tumor epithelial cells and fibroblasts within the TME of COKC. Clinical data were also incorporated to identify key clinical features associated with COKC. This work not only enhances our understanding of the cellular architecture of COKC but also provides valuable insights to advance diagnostic, therapeutic, and molecular research related to COKC.

## 2 Materials and methods

### 2.1 Tumor specimen collection

Three samples diagnosed with malignant transformation of OKC to squamous cell carcinoma were collected from The Department of Head and Neck Oncology, West China Hospital of Stomatology, Sichuan University. The diagnosis of COKC requires confirmation through simultaneous microscopic observation of characteristic OKC lesions along with transitional areas exhibiting malignant transformation into squamous cell carcinoma. The diagnosis of each case was confirmed by an experienced pathologist. None of the patients had undergone radiation, chemotherapy, or any other antitumor treatments prior to surgery.

### 2.2 Clinical data collection

In this retrospective study, we reviewed the clinicopathologic records of 13 patients with OKC who underwent malignant transformation into intraosseous squamous cell carcinoma, along with follow-up evaluations. Additionally, 277 OKC patients were included in the control group. Data were collected from inpatients admitted to The Department of Head and Neck Oncology, West China Hospital of Stomatology, Sichuan University, between January 1, 2014, and January 1, 2024.

### 2.3 Tissue processing

Tissue specimens were placed on ice to allow sedimentation to the bottom of the container. They were then washed with 1 × PBS to remove the preservative solution and blood from the tissues. A digestion mixture containing 1 mg/ml neutral protease, 1 mg/ml collagenase types I, II, and IV, along with DNase I, was prepared. The tissue was finely minced using scissors and incubated at 37 °C with agitation at 160 rpm for approximately 30 min to facilitate digest. Following digestion, the cell suspension was filtered through a 40 μm cell strainer, centrifuged, and the cell pellet was resuspended in 3 ml of red blood cell lysis buffer. The suspension was incubated at 4 °C for 5 min to lyse any remaining red blood cells. The reaction was stopped, followed by centrifugation. A 10 μl sample of the cell suspension was taken for trypan blue staining and microscopic examination.

### 2.4 Single-cell Library preparation and sequencing

Following the manufacturer's protocol, we encapsulated single cells into droplets using the Chromium Single Cell 3′ Library & Gel Bead and Multiplex Kit (10x Genomics, version 3.1) to construct scRNA-seq libraries. Cell suspensions with viability exceeding 90 % were loaded into the Chromium Single Cell Controller (10x Genomics) to generate single-cell gel beads in emulsion. Subsequent procedures included reverse transcription cleanup, cDNA amplification, and library quality assessment. The scRNA-seq libraries were sequenced on the Illumina NovaSeq 6000 platform to generate 150-bp paired-end reads. Raw gene expression matrices for each sample were generated by aligning the reads to the human genome (hg38) using CellRanger (10x Genomics).

### 2.5 Preprocessing of scRNA-seq Data

Expression matrices from all samples were processed into Seurat objects using Seurat (version 4.3.0) [15]. The data underwent stringent quality control (QC) filtering to ensure that only high-quality cells were included in the analysis. Cells with fewer than 200 genes or more than 5000 genes were excluded to remove low-complexity cells, potential doublets, and cells with high transcriptional complexity. Cells with more than 20 % mitochondrial gene content were discarded, as elevated mitochondrial content is often indicative of cell stress or death. Doublets were identified and removed using the DoubletFinder algorithm (version 2.0.3), which predicts doublet cells based on gene expression profiles, minimizing the inclusion of artificial mixed cell populations. The Seurat built-in regression method was applied to remove several sources of technical variation, specifically cell cycle scores ("S.Score" and "G2M.Score"), RNA content ("nCount_RNA"), and mitochondrial gene content ("percent.mt"). This ensured that observed gene expression differences were not confounded by these technical factors. After these regression steps, the data were prepared for clustering, differential expression analysis, and other downstream applications, retaining a total of 22,509 cells for further analysis.

### 2.6 Integration, dimensionality reduction, clustering, and cell annotation

We obtained scRNA-seq data for three OKC samples from GSE176351 and six OSCC samples from GSE172577 for integrative analysis [16], [17]. To address batch effects across different patients, we used Harmony (version 1.1.0) for batch correction [18]. Initially, expression matrices were normalized using the NormalizeData function in the Seurat package (version 4.3.0). The FindVariableFeatures function was then used to identify the top 2000 variable genes for subsequent principal component analysis. Data scaling was performed with "ScaleData", and the first 50 principal components of the expression matrix were analyzed using "RunPCA". Subsequently, clustering was performed using the first 25 principal components with the 'FindNeighbors' and 'FindClusters' functions, applying a resolution of 0.4. The resulting clusters were visualized using two-dimensional uniform manifold approximation and projection (UMAP). Characteristic genes for each cell subgroup were identified utilizing the "FindAllMarkersMAESTRO" function in the MAESTRO package (version 1.5.1) [19]. Genes with logFC > 1.0 and padjust < 0.05 were considered as marker genes for each subgroup. Manual annotation of each cell subgroup was performed by cross-referencing these characteristic genes with the CellMarker 2.0 and PanglaoDB databases [20], [21].

### 2.7 Tissue microarray (TMA) preparation

The tissue microarray consisted of 76 OSCC tissue samples, sourced from The Department of Head and Neck Oncology, West China Hospital of Stomatology, Sichuan University. The array was constructed by SHANGHAI XINCHAO BIOTECH Co.

### 2.8 H&E and immunohistochemical Staining

The tumor tissues were fixed in a 4 % paraformaldehyde solution (BL539A, Biosharp, Guangzhou, China), embedded in paraffin, and then processed for hematoxylin and eosin (H&E) staining. Immunohistochemical staining was performed following the manufacturer's protocol, with the Ki67 antibody (ab15580, Abcam), the S100 antibody (RAB-0150, Fuzhou Maixin Biotech) and the MAST4 antibody (ER63917, HUABIO). The staining results were imaged using the Aperio ScanScope FL+GL system (Leica, Buffalo Grove, USA). For multiplex immunohistochemistry (mIHC), a triple-marker, four-color multiplex fluorescence staining kit (AFIHC024, Aifang Biotechnology) was utilized following the manufacturer’s instructions. The primary antibodies used were COL1A1 (A16891, Abclonal), ID2 (DF6178, Affinity), PRKG1 (21646-1-AP, Proteintech), CD68 (25747-1-AP, Proteintech), IL1B (YT5201, Immunoway), and CD206 (81525-1-RR, Proteintech). Imaging was performed using a SpinSR confocal microscope (OLYMPUS, Tokyo, Japan).

### 2.9 Cell stemness analysis

The CytoTRACE scores for epithelial cells were calculated using the R package CytoTRACE (version 0.3.3) [22]. CytoTRACE scores range from 0 to 1, with higher scores indicating greater stemness.

### 2.10 Pathway metabolic activity analysis

The scMetabolism package (version 0.2.1) was utilized for quantifying and visualizing the metabolic characteristics of epithelial cells at single-cell resolution [23].

### 2.11 GO and KEGG enrichment analysis

Gene Ontology (GO) enrichment and Kyoto Encyclopedia of Genes and Genomes (KEGG) analysis for upregulated differentially expressed genes (DEGs) within a cell subtype were performed using the "enrichGO" and "enrichKEGG" functions of the clusterProfiler package (version 4.9.0) [24]. Adjusted p-values were calculated using hypergeometric distribution and Benjamini-Hochberg correction, with statistical significance set at p < 0.05.

### 2.12 InferCNV and clonality analysis

The inferCNV package (version 1.12.0) was used to analyze the copy number variation (CNV) patterns in COKC cells. Default parameters were applied, with epithelial cells from OKC serving as the reference for estimate CNV in the malignant cells. Subsequently, a clonality tree was constructed using the UPhyloplot2 algorithm (version 2.3) to investigate subclonal patterns in COKC cells [25].

### 2.13 Gene set variation analysis (GSVA)

The GSVA package (version 1.44.5) was used to assign pathway activity estimates to individual cells based on scRNA-seq data [26]. Pathways used in the enrichment analysis, particularly the Hallmark gene sets, were obtained from the MSigDB database [27].

### 2.14 Gene set enrichment analysis (GSEA)

The "gseKEGG" function of the clusterProfiler package (version 4.9.0) was used to identify functional pathway states in neuronal subpopulations.

### 2.15 Gene interaction network analysis

Functional networks and gene linkage data were extracted using the STRING database [28]. Subsequently, connectivity data from STRING were analyzed using the igraph package (version 1.3.4). Network analysis of the extracted connectivity data was performed using edge betweenness and random walk methods.

### 2.16 Intercellular communication analysis

CellChat (version 1.4.0) was used to explore potential cell-cell interactions within the COKC TME, using default parameters and the ligand-receptor pair database [29].

### 2.17 PySCENIC analysis

The PySCENIC workflow (version 0.9.1) with default parameters was used to identify gene regulatory networks (GRNs) and the activity of transcription factors (TFs) [30].

### 2.18 Pseudotime trajectory analysis

The Slingshot R package (version 2.6.0) was used to elucidate the developmental trajectories of tumor epithelial cells [31]. We defined the "C1" subgroup characterized by the lowest degree of copy number variations as the starting point of cellular differentiation and "keratinocytes" as the terminally differentiated cells.

### 2.19 TCGA analysis

Gene expression profiles and clinical data from TCGA-HNSC patients were retrieved using the TCGAbiolinks R package (version 2.25.3) [32]. Gene sets listed in Table S3 were used to compute GSVA scores for each sample as input. The "surv_cutpoint" function in the survminer R package (version 0.4.9) was used to determine the cutoff criteria.

### 2.20 Statistics

All statistical analyses and data visualizations were performed using R software (version 4.1.3). A significance level of *P* < 0.05 was considered statistically significant for the analysis.

## 3 Results

### 3.1 Single-cell expression atlas and cell type identification in COKC TME

**Fig. 1:**
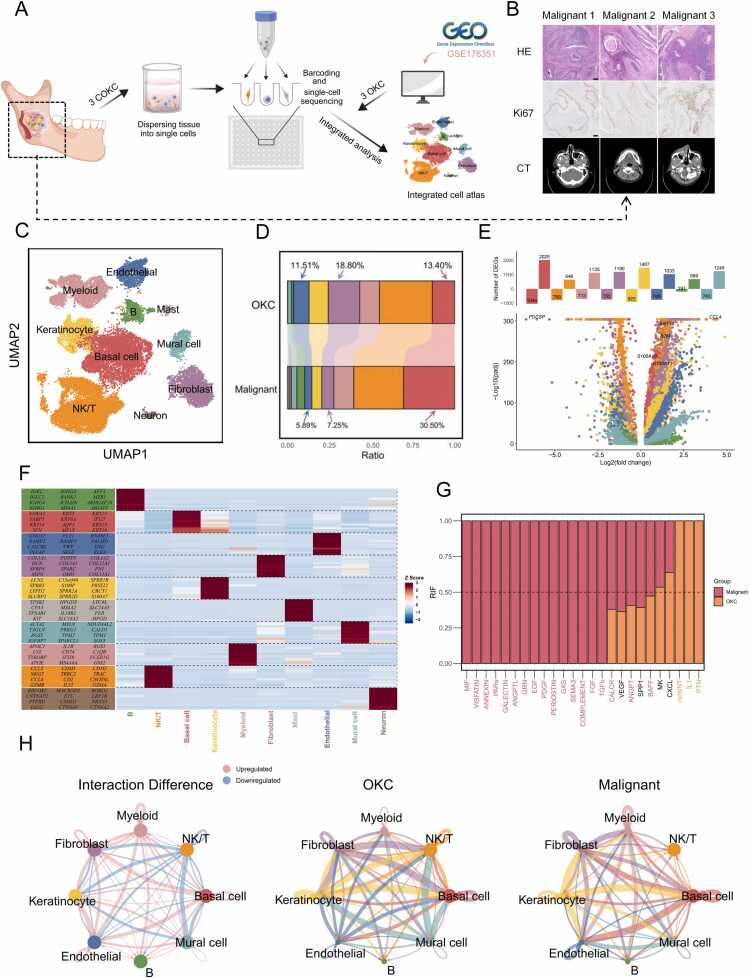
Single-cell expression atlas and cell type identification in COKC TME. (A) The workflow illustrates the sample collection, single-cell suspension preparation, single-cell sequencing, and bioinformatics-based analysis process. (B) H&E staining, Ki67 immunohistochemistry, and imaging examinations display characteristics of all COKC cases (scale bars, 200 µm). (C) UMAP plots of 22,509 cells based on analysis of 10x Genomics scRNA-seq data, revealing the major cell types within COKC and OKC tissues. (D) Stacked histograms showing the percentage of 10 cell types out of all cells. (E) Volcano plots and bidirectional bar charts display the distribution (bottom) and number of differential genes (top) in each cell subpopulation. (F) Heatmap displays the top genes for each cell subpopulation. (G) Relative information flow (RIF) compares signaling pathways between COKC and OKC. (H) Interaction networks among cell types in OKC and COKC. Pink lines indicate upregulated interactions, blue lines represent downregulated interactions. Basal cells dominate malignant interactions.

We collected tumor tissues from three COKC patients for scRNA-seq (Fig. 1A). Pathological examination along with imaging confirmed that these samples exhibited typical features of COKC (Fig. 1B). Additionally, we performed a comparative analysis by integrating scRNA-seq data from three publicly available OKC samples [16]. After applying a single-cell analysis pipeline to remove low-quality cells, a total of 22,509 single cells were successfully sequenced. Among them, 14,479 cells (64.3 %) were derived from COKC patients, while 8030 cells (35.7 %) originated from OKC patients. We identified ten major cell types, including basal cells, keratinocytes, myeloid cells, NK/T cells, plasma cells, mast cells, fibroblasts, endothelial cells, pericytes, and neuronal cells (Fig. 1C and Table S1). Significant differences were observed in the distribution of cell types between COKC and OKC samples (Fig. 1D, S1A, S1B and S1C). In COKC, the basal cell subtype exhibits the highest number of upregulated differentially expressed genes. Among non-epithelial cells, fibroblasts and pericytes show the greatest gene upregulation (Fig. 1E). The heatmap illustrates the top genes for each cellular subtype (Fig. 1F). Intercellular communication within cell clusters was analyzed using CellChat [29]. In the TME, 18 pathways, including MIF, VISFATIN, and ANNEXIN, a exhibited relatively increased information flow in COKC (Fig. 1G). Furthermore, interactions between fibroblasts and basal cells were significantly elevated (Fig. 1H).

### 3.2 Tumor cell transcriptional heterogeneity in COKC

**Fig. 2:**
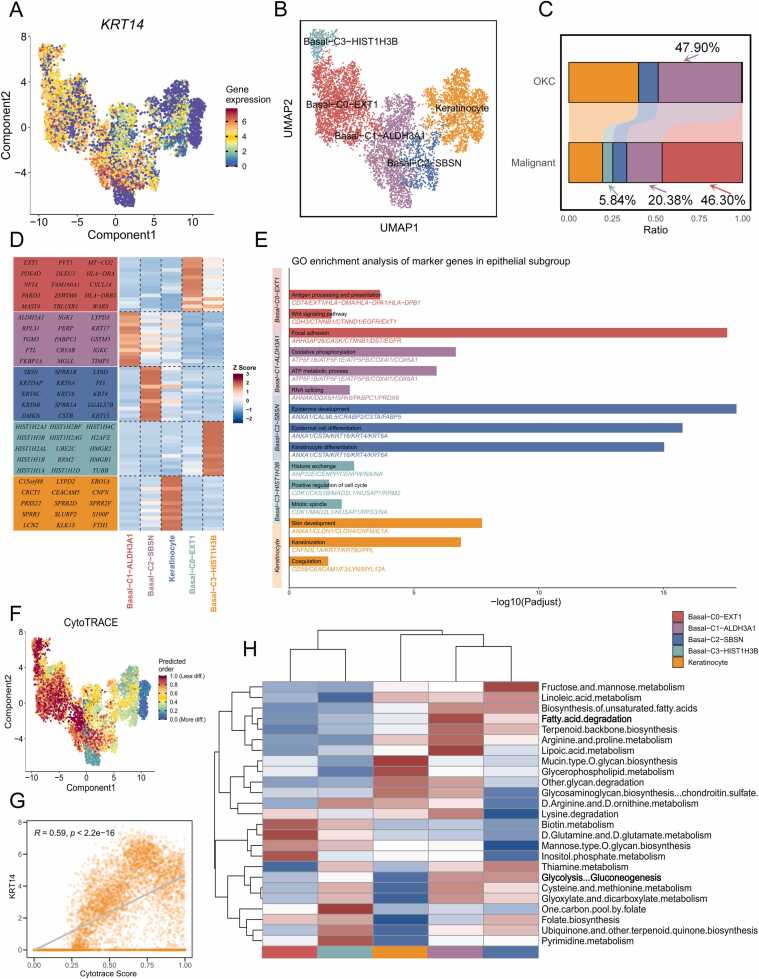
Transcriptomic heterogeneity analysis of COKC tumor epithelial cell subpopulations. (A) UMAP plot illustrating the expression level of KRT14. (B) UMAP plot demonstrating the clustering of tumor epithelial cell subpopulations. (C) Stacked histogram showing the percentage of tumor epithelial cell types from COKC and OKC tissues in the total epithelial cell population. (D) Heatmap displays the top genes for each epithelial cell subpopulations. (E) GO enrichment analysis of characteristic genes for each epithelial cell subgroup. (F) UMAP plot depicting the distribution of CytoTRACE scores in tumor epithelia. Dark green indicates lower scores (lower stemness), and dark red indicates higher scores (higher stemness). (G) Correlation between the expression level of KRT14 in tumor epithelia and CytoTRACE scores. (H) Heatmap displaying the differences in expression scores of metabolic pathways across different epithelial subgroups.

Keratin 14 (KRT14), a marker of human basal cells, was found to be expressed in the vast majority of tumor cells (Fig. 2A) [33], [34]. To characterize the distribution of epithelial subtypes, we reclassified 7096 epithelial cells into four basal cell subgroups and one keratinocyte subgroup (Fig. 2B and Table S2). Notably, the Basal-C0-EXT1 and Basal-C3-HIST1H3B subgroups were exclusively identified in COKC, suggesting their potential pivotal role in driving the malignant transformation of OKC (Fig. 2C, S1D and S1E). The top genes of each epithelial cell subtype are illustrated in the heatmap (Fig. 2D). Enrichment analyses revealed that the keratinocyte subgroup exhibited characteristics of keratinization (Fig. 2E). The C0 subtype showed upregulation of antigen presentation and focal adhesion functions. The C1 subtype exhibited increased oxidative phosphorylation and ATP metabolism processes. The C2 subgroup, defined by high SBSN expression and traits linked to epidermal and keratinocyte development, may represent precursors to keratinocytes [35], [36]. Additionally, the COKC-specific C3 subgroup exhibited upregulation of histone-related genes, along with enhanced histone exchange and positive regulation of cell cycle.

We demonstrated heterogeneity in stemness and differentiation states among tumor cells, with the COKC-specific epithelial C0 and C3 subgroups exhibiting the highest stemness levels. This suggests a potential dedifferentiation process in OKC carcinogenesis (Fig. 2F). Consistent with findings in other cancer types, KRT14 expression in COKC is closely associated with cell stemness (Fig. 2G) [33]. Additionally, the high-energy metabolic state of C1 subgroup cells was validated, particularly highlighting fatty acid metabolism and glucose utilization (Fig. 2H).

### 3.3 Tumor cell copy number variation and transcriptional regulation analysis in COKC

**Fig. 3:**
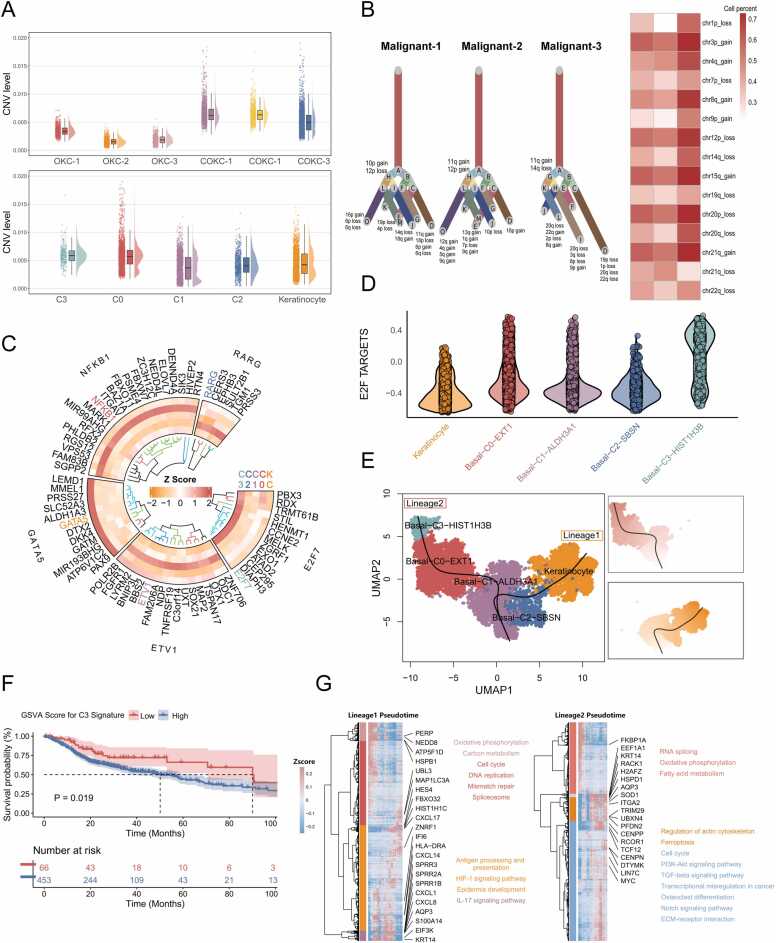
Copy number variation levels, transcriptional regulation, and differentiation trajectories in COKC. (A) Raincloud plots comparing the differences in copy number variation levels between different samples (top) and among different epithelial cell subpopulations (bottom). (B) A clonal evolution tree generated using the UPhyloplot2 algorithm displays distinct CNV patterns in cells from three COKC tumors (left), alongside a heatmap showing shared copy number events among individual samples (right). (C) Circular heatmap displaying the most significantly activated TF and the expression levels of their downstream target genes in each epithelial subgroup. (D) Comparison of E2F target pathway scores among different epithelial cell subgroups. (E) Evolutionary trajectories of all COKC tumor epithelial cells inferred through Slingshot. (F) Kaplan-Meier survival curves for TCGA HNSC patients, categorized based on high or low GSVA scores of gene sets characteristic of the C3 subgroup. (G) Differential expression genes along pseudotime in two distinct differentiation pathways are clustered into four subclusters. The most important annotated GO terms and KEGG pathways are provided for each cluster.

CNV levels in COKC epithelial cells were significantly higher than those in OKC. When mapping CNV scores to different epithelial subgroups, we found that the C0 and C3 subgroups exhibited the highest CNV levels (Fig. 3A and S1F). Moreover, variations in evolutionary tree nodes were observed across different COKC patients. However, some shared CNV events were identified, including gains in Chr3p, loss in Chr12p, and gain in Chr15q. These common subclonal CNV events may serve as the foundation of OKC malignancy (Fig. 3B).

The expression profiles of the most highly activated transcription factors and their downstream target genes in each subgroup are illustrated (Fig. 3C and S2A). Notably, the transcription factor E2F7 is highly activated within the C3 subgroup. Supporting this, GSVA scoring confirms the enrichment of the E2F target pathway in this subgroup, suggesting that E2F signaling activation may underlie the malignant phenotype of the C3 subgroup (Fig. 3D and S2A).

### 3.4 Transcriptional trajectory analysis during OKC carcinogenesis

Two distinct differentiation pathways were identified (Fig. 3E). One pathway represents normal OKC epithelial differentiation, progressing from ALDH3A1+ basal cells to SBSN+ basal cells and ultimately leading to keratinocyte formation. The other pathway, characterized by the transition from C0 to C3, is likely involved in malignant development. Notably, the C3 subgroup exhibited significant upregulation of histone-related genes (e.g., HIST1H2AJ, HIST1H3B, HIST1H1B, and HIST1H1A) (Fig. S2B and Table S3). Given that most COKCs progress to jaw squamous cell carcinoma, we performed a survival analysis on highly expressed marker genes in the C3 subgroup using the TCGA head and neck squamous cell carcinoma (HNSC) dataset. The results suggest that C3 subgroup characteristics are strongly associated with clinical progression and survival outcomes in HNSC. (Fig. 3F and S2C).

Aquaporin 3 (AQP3) is implicated in the formation of cystic lesions and consistently maintains high expression levels throughout the malignant transformation pathway (Fig. S2D) [37]. The expression changes of KRT14 further support the dedifferentiation trend in COKC. Additionally, differences in signaling pathway dynamics were observed between the two differentiation pathways (Fig. 3G). Notably, pathways such as the HIF-1 signaling pathway, which is known to be involved in OKC formation and development, were significantly upregulated in the OKC differentiation lineage [38]. In contrast, pathways associated with cell development (cell cycle, NOTCH, transcriptional dysregulation) and cancer invasion and metastasis (TGF-β, PI3K-AKT, ECM, and osteoclastogenesis) were significantly upregulated during the lineage differentiation process of OKC malignant transformation.

### 3.5 Fibroblast phenotypic transition in OKC carcinogenesis

**Fig. 4:**
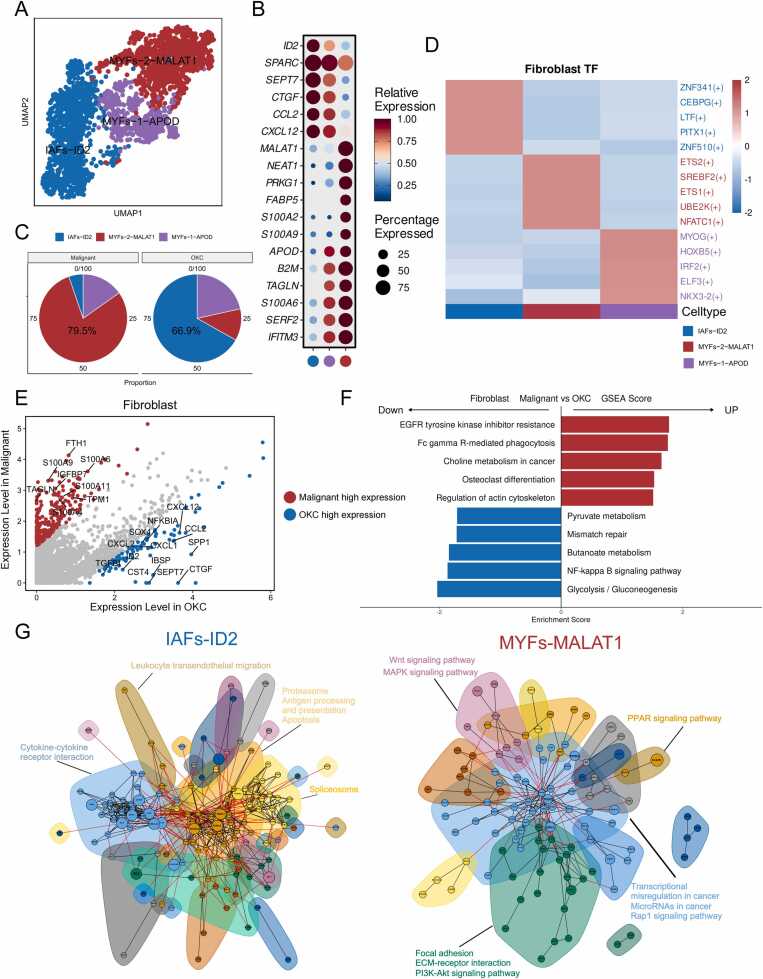
Evaluating the functional state of fibroblasts in COKC. (A) UMAP plot illustrating the clustering of fibroblast subpopulations. (B) The dot plot displays the top genes for each fibroblast subtype. (C) Pie charts show the proportions of different types of fibroblasts in COKC and OKC. (D) Heatmap displays differences in TF activity among fibroblast subpopulations. (E) Volcano plot illustrates differential genes between fibroblasts derived from COKC and OKC. (F) Significant regulated pathways derived from GSEA analysis. Red and blue bars represent pathways that are upregulated and downregulated, respectively, in fibroblasts from COKC. (G) Gene interaction network for characteristic genes within the IFBs-ID2 (left) and MFBs-2-MALAT (right) subpopulations.

Fibroblasts are the predominant stromal cells in COKC (Fig. 1D). We reclassified a total of 4205 fibroblasts, including 2291 (54.5 %) from COKC and 1914 (45.5 %) from OKC tissues (Fig. 4A and Table S4). These fibroblasts exhibited significant heterogeneity, and were categorized into three main subtypes: inflammation-associated fibroblasts (IFBs-ID2), myofibroblasts 1 (MFBs-1-APOD), and myofibroblasts 2 (MFBs-2-MALAT1), with myofibroblasts 1 potentially representing a transitional state (Fig. 4B and S2E). Myofibroblasts accumulated in the COKC stroma, whereas inflammation-associated fibroblasts predominantly originated from OKC tissues (Fig. 4C and S2F). These findings were confirmed by mIHC staining images (Fig. S2G).

ETS1 and ETS2 were significantly upregulated in the MFBs-2-MALAT1 subgroup (Fig. 4D). Differential gene analysis revealed a significant upregulation of the S100A protein family and myofibroblast markers in COKC, whereas the expression of inflammation-related factors decreased, consistent with the cell classification results (Fig. 4E). Moreover, GSEA identified significant upregulation of pathways related to osteoclast differentiation and actin cytoskeleton regulation in fibroblasts within COKC (Fig. 4F). Additionally, protein interaction network analysis was performed for IFBs-ID2 and MFBs-2-MALAT1 subgroups. Subnetwork analysis based on functional annotation revealed that IFBs-ID2 primarily influences antigen presentation, apoptosis, and cytokine interactions, whereas the predominant MFBs-2-MALAT1 subgroup in COKC is significantly associated with tumor invasion and migration pathways, such as MAPK, PI3K-AKT, and Wnt signaling (Fig. 4G).

### 3.6 Macrophage phenotypic transformation in OKC carcinogenesis

Myeloid cells undergo the most significant changes among immune cell populations following the malignant transformation of OKC (Fig. 1E). We identified several myeloid subpopulations based on specific markers, including Macrophage-APOE, Macrophage-C1QA, Macrophage-IL1B, Macrophage-CD209, Macrophage-MKI67, Macrophage-IDO1, Neutrophils, cDC-CD1C, cDC-LILRA4, cDC-CLEC9A, and cDC-LAMP3 (Fig. S3A, S3B, and Table S5). Macrophage-APOE and Macrophage-C1QA were highly enriched in COKC, whereas Macrophage-IL1B and Macrophage-CD209 were predominantly found in OKC (Fig. S3C). Further enrichment analysis revealed that Macrophage-APOE is strongly associated with antigen presentation and the positive regulation of lymphocyte activation, whereas Macrophage-IL1B is linked to interleukin-2 production and tumor necrosis factor superfamily cytokine production (Fig. S3D).

Macrophage-IL1B is classified as a prototypical M1 macrophage [39], [40]. These findings suggest a potential transition between M1 and M2 macrophages during OKC carcinogenesis. We compiled marker genes for M1 and M2 macrophages and performed gene set scoring, revealing that M2 macrophages underwent significant changes during OKC carcinogenesis (Fig. S3E and Table S6). This transition is characterized by the downregulation of IL1B, CCL2, and PTGS2, alongside the upregulation of CD163, MRC1, and CHI3L1 (Fig. S3F). This finding was validated through mIHC staining of CD68, IL1B, and CD206 (Fig. S3G). Furthermore, we investigated macrophage-endothelial cell interactions and found that Macrophage-APOE, which is highly enriched in COKC, promotes angiogenesis by interacting with endothelial cells. This interaction is mediated through various ligand-receptor pairs, including VEGFA-VEGFR1 and VEGFB-VEGFR1 (Fig. S3H). Collectively, these findings suggest that during OKC carcinogenesis, macrophages transition from M1 to M2 phenotypes, potentially facilitating tumor progression by promoting angiogenesis through interactions with endothelial cells.

### 3.7 Strong neuroinvasive characteristics of tumors in COKC

**Fig. 5:**
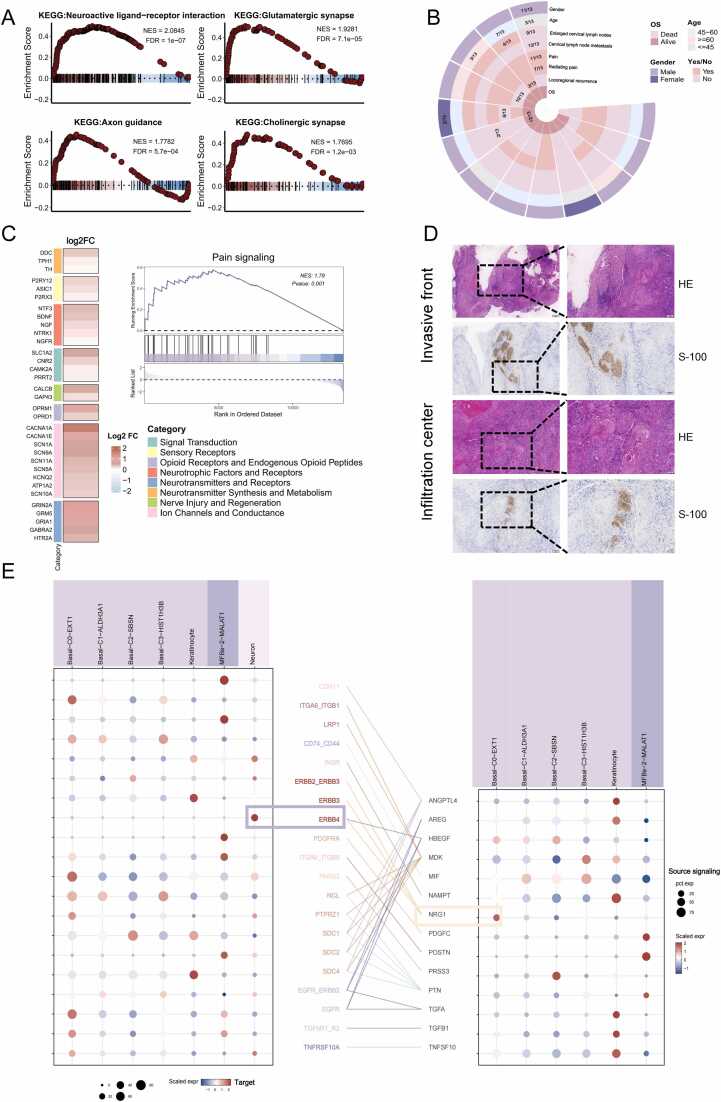
Neuroaggressiveness and clinical data of COKC. (A) GSEA enrichment analysis of marker genes in nerve cell subsets. (B) Clinical data and follow-up information of 13 COKC patients in our institution. (C) Expression of Pain Signaling-Related Molecules in Neuronal Subpopulations (left) and GSEA Analysis of Pain Signaling Pathway (right). (D) H&E and immunohistochemical staining reveal neural invasion and infiltration in COKC samples. (E) The dot plot shows the gene expression levels of receptor-ligand pairs involved in interactions between different clusters in COKC.

A notable finding in our study was the presence of a unique neuronal cell subpopulation within COKC tumors (Fig. 1C and D). GSEA enrichment analysis of the marker genes for these neuronal subgroups confirmed the accuracy of the cell classification (Fig. 5A). The presence of neuronal cells within COKC tumor samples suggests potential involvement of the nervous system and indicates strong neuroinvasive characteristics exhibited by COKC lesions. To further investigate this, we reviewed 13 cases diagnosed with COKC that underwent surgical treatment at our institution over the past decade. Clinical features, treatment approaches, and patient outcomes were summarized (Fig. 5B and Table 1). The radiographic features of COKC patients under different imaging modalities are presented in Fig. S4A. Among the findings, 11 patients reported pain in the lesion area, with more than half describing radiating pain in the ipsilateral facial region. Additionally, we compared the clinical characteristics of these COKC patients with those of 277 OKC patients and found that COKC patients exhibited significantly more pronounced pain symptoms (Table 2). This findings suggest that COKC patients may experience typical cancer-associated pain due to tumor invasion into nerves. In a representative COKC case, head and neck MRI revealed disease progression extending along the rupture foramen, invading surrounding tissues, the trigeminal ganglion, and eventually entering the cranium. This highlights COKC's propensity for invading nerve channels and the central nervous system (Fig. S4B).

**Table 1:** Clinical and prognostic information of 13 COKC patients.

Number	Gender	Age	Region of lesion	Enlarged cervical lymph nodes	Cervical lymph node metastasis	Pain	Radiating pain	Gorlin syndrome	Final option of treatment					Prognosis			
									Jawbone resection	Neck dissection	Tissue repair	Radiation therapy	Chemotherapy	Locoregional recurrence	LRRFS.time (days)	OS	OS.time(days)
1	Male	55	Right maxilla	No	No	Yes	Yes	Yes	No	No	No	Yes	Yes	No	126	Dead	126
2	Male	59	Left mandible	No	No	Yes	Yes	No	Yes	No	No	No	No	No	3274	Alive	3274
3	Male	78	Right mandible	No	No	Yes	No	No	Yes	No	No	Yes	No	No	1089	Alive	1089
4	Female	35	Right mandible	No	No	No	No	Yes	Yes	Suprahyoid	Fibula	No	No	No	712	Alive	712
5	Male	49	Right maxilla	No	No	Yes	Yes	No	Yes	No	Anterolateral thigh	Yes	Yes	Yes	180	Alive	284
6	Male	52	Bilateral maxilla	No	No	Yes	No	No	No	No	No	Yes	Yes	No	275	Alive	275
7	Male	45	Left mandible	No	No	Yes	No	No	Yes	No	Fibula	No	No	No	198	Alive	198
8	Male	58	Bilateral mandible	No	No	No	No	Yes	Yes	No	Rib	No	No	No	3713	Alive	3713
9	Female	57	Left mandible	Yes	No	Yes	No	No	Yes	No	No	No	No	Yes	149	Alive	1091
10	Male	65	Left mandible	Yes	No	Yes	Yes	No	Yes	Suprahyoid	Fibula	No	No	No	46	Alive	46
11	Male	64	Bilateral maxilla	Yes	No	Yes	Yes	No	Yes	No	Anterolateral thigh	No	No	No	36	Alive	36
12	Male	55	Left mandible	Yes	No	Yes	Yes	No	Yes	No	Latissimus dorsi	No	No	Yes	476	Alive	598
13	Male	32	Right maxilla	No	No	Yes	Yes	No	Yes	Suprahyoid	Anterolateral thigh	No	No	No	50	Alive	50

OS: overall survival; LRRFS: Locoregional recurrence-free survival

**Table 2:** Comparative analysis of clinical features between COKC and OKC.

Characteristic	Total (n = 290)	COKC (n = 13)	OKC (n = 277)	Statistic	*P*	SMD
Age, Mean ± SD	39.51 ± 17.23	54.15 ± 12.26	38.82 ± 17.14	t = 3.185	**0.002**	−0.895
Gender, n (%)				χ² = 5.897	**0.015**	
male	150 (51.72)	11 (84.62)	139 (50.18)			−0.689
female	140 (48.28)	2 (15.38)	138 (49.82)			0.689
Lesion number, n (%)				χ² = 2.152	0.142	
solitary	266 (91.72)	10 (76.92)	256 (92.42)			0.585
multiple	24 (8.28)	3 (23.08)	21 (7.58)			−0.585
Infection history, n (%)				χ² = 1.517	0.218	
no	188 (64.83)	11 (84.62)	177 (63.90)			−0.431
yes	102 (35.17)	2 (15.38)	100 (36.10)			0.431
Recurrence, n (%)				χ² = 1.880	0.170	
no	170 (58.62)	10 (76.92)	160 (57.76)			−0.388
yes	120 (41.38)	3 (23.08)	117 (42.24)			0.388
Pain, n (%)				χ² = 22.702	**< .001**	
no	217 (75.09)	2 (15.38)	215 (77.90)			1.507
yes	72 (24.91)	11 (84.62)	61 (22.10)			−1.507
Gorlin syndrome, n (%)				χ² = 2.152	0.142	
no	266 (91.72)	10 (76.92)	256 (92.42)			0.585
y es	24 (8.28)	3 (23.08)	21 (7.58)			−0.585

The statistical results. *P* < 0.05 difference was statistically significant.

Further analysis revealed that the neuronal subset unique to COKC exhibited significant upregulation of pain signaling pathways and numerous pain-related molecules (Fig. 5C). Additionally, HE staining and immunohistochemical analysis clearly demonstrated nerve invasion and infiltration by COKC (Fig. 5D). Analysis of tumor epithelial cell–neuronal cell interactions revealed that neuronal cells exhibited the highest expression of the ERBB4 receptor, with HBEGF and NRG1 identified as the most likely tumor-derived ligands. (Fig. 5E).

### 3.8 Low potential for cervical lymph node metastatic in COKC

**Fig. 6:**
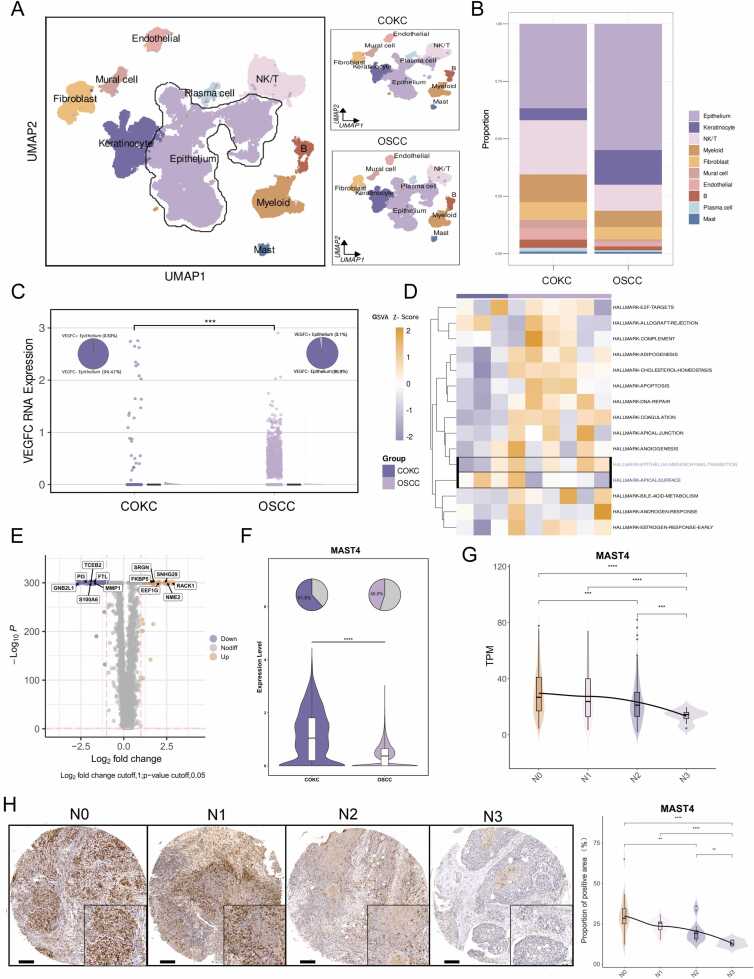
Integration analysis of COKC and OSCC reveals low lymphatic metastasis potential. (A)UMAP maps reveal the main cell types and sample sources in COKC and OSCC tissues. (B) The accumulation histogram shows the percentage of all cells from the 10 cell types from COKC and OSCC tissues. (C) Differences in VEGFC gene expression and proportion of positive cells in COKC and OSCC tumor epithelial cells (***, *P* < 0.001). (D) Differential heatmap of GSVA scoring hallmark gene sets in COKC and OSCC samples. (E) The volcano plot illustrates the differentially expressed genes between COKC and OSCC. (F) The violinplot depicted the differential expression of the MAST4 gene between COKC and OSCC. (G) Expression levels of MAST4 across different lymph node metastasis stages in TCGA-HNSC cohort. (H) Immunohistochemical staining of MAST4 across different lymph node metastasis stages (left) and quantitative analysis (right) in OSCC tissue microarray.

We collected a series of COKC cases from multiple institutions. In this dataset, which includes 13 cases from our institution, none of the 25 COKC patients exhibited cervical lymph node metastasis (Fig. S5A) [6], [9], [41], [42]. Lymph node metastasis (LNM) is a significant adverse prognostic factor in oral squamous cell carcinoma (OSCC), occurring in approximately 40 % of cases [43]. To investigate the low lymph node metastatic potential of COKC, we compared single-cell transcriptomic data from three COKC cases and six OSCC cases [17]. After excluding low-quality cells, we identified 10 cell subpopulations, including epithelial and keratinocyte subgroups (Fig. 6A, S5B and Table S7). COKC samples exhibited a higher abundance of immune cells, particularly T cells, suggesting a more active immune microenvironment (Fig. 6B). Further analysis revealed lower VEGFC expression in COKC tumor cells, a factor associated with lymphatic dilation, and reduced EMT activity (Fig. 6C and D) [44]. These findings suggest potential mechanisms underlying the low lymph node metastatic potential in COKC, including an active immune microenvironment, decreased VEGFC expression, and reduced EMT activity.

Furthermore, Microtubule Associated Serine/Threonine Kinase Family Member 4 (MAST4) was identified as a potential key molecule contributing to the low lymphatic metastatic capacity of COKC. As shown in Figure S5C, MAST4 was identified through a stepwise screening process, including single-cell differential expression analysis (Fig. 6E and F), TCGA-HNSC bulk RNA-seq survival analysis (Fig. S5D), univariate Cox regression (Fig. S5E), and N-stage correlation studies (Fig. 6G). MAST4 is highly expressed in the tumor epithelium of COKC but exhibits low expression in OSCC (Fig. S5F). In OSCC, MAST4 is associated with favorable prognosis and lower lymph node metastasis staging. These observations were further validated by immunohistochemical analysis of 76 OSCC TMA samples (Fig. 6H). Therefore, MAST4 may contribute to the low lymphatic metastatic potential of COKC.

## 4 Discussion

In this study, single-cell sequencing was employed to comprehensively characterize the malignant transformation process of OKC. By integrating clinical data, we further uncovered previously underrecognized clinical features of COKC, particularly its strong neuroinvasive potential and low lymph node metastatic potential (Fig. S5G).

Our study revealed the presence of multiple tumor cell subpopulations with distinct functions in COKC. The Basal-C0-EXT1 and Basal-C3-HIST1H3B subpopulations are unique to COKC, suggesting that these subpopulations may play critical roles in driving the malignant transformation of OKC. Enrichment analysis showed that the C3 subpopulation is associated with cell cycle progression and mitotic division, while the C0 subpopulation is linked to adhesive plaques. This suggests that the C3 and C0 subpopulations have distinct characteristics, with the C3 subpopulation exhibiting the highest tumor stemness and the C0 subpopulation displaying the strongest invasive potential. The C2 subpopulation showed high expression of SBSN and displayed features characteristic of epidermal and keratinocyte development, suggesting that these cells may represent keratinocyte precursors. Previous studies have suggested that SBSN serves as an early marker of differentiation in undifferentiated keratinocytes, consistent with our findings [35], [36].

Extensive cancer research has shown that during carcinogenesis, normal regulatory mechanisms of cell renewal and differentiation become overactivated, leading to a gradual restoration of cellular stemness [45], [46]. We identified a malignant differentiation pathway distinct from that of OKC. This pathway involves cellular dedifferentiation, with activation of the E2F signaling pathway as a key driver. In the Basal-C3-HIST1H3B subpopulation, tumor cells exhibited a highly stem-like state with sustained E2F pathway activation. Previous studies have demonstrated the critical role of the E2F pathway in regulating tumor cell stemness. E2F family transcription factors primarily regulate the G1/S phase transition in normal cells, ensuring timely entry into the DNA replication phase [47]. In tumor cells, overactivation of the E2F pathway frequently results from Rb protein inactivation or upstream signaling abnormalities. Such overactivation leads to cell cycle dysregulation, accelerating tumor cell proliferation and promoting stemness characteristics through upregulating stemness-related genes, including Nanog, Sox2, and Oct4 [48], [49]. Stemness characteristics include enhanced self-renewal capacity, preserved differentiation potential, and resistance to chemotherapy and radiotherapy, enabling tumor stem cells to survive treatment and drive recurrence and metastasis. A retinoblastoma study found that elevated E2F levels disrupted cell cycle regulation, triggering a cancerous phenotype [50]. The E2F signaling pathway is also recognized as a prognostic indicator in various cancers, including breast, lung, ovarian, nasopharyngeal, oropharyngeal, and hepatocellular cancers [49], [51]. In head and neck squamous cancer studies, E2F mRNA expression was upregulated compared to adjacent tissues, consistent with E2F pathway activation in recurrent OSCC after radiotherapy [52]. In this study, the high E2F signaling activity observed in the C3 subpopulation suggests that targeting E2F to inhibit its regulation of stemness genes could be a potential strategy for treating COKC and preventing recurrence.

Chromatin reprogramming plays a crucial role in the dedifferentiation of cancer cells, as an “open” chromatin state promotes the maintenance of cellular pluripotency [53]. The significant dedifferentiation observed in the C3 subpopulation aligns with the upregulation of histone exchange and histone binding levels. Previous studies have shown that inflammation in pancreatic acinar cells not only drive cellular dedifferentiation but also lead to stem cell damage and impaired differentiation capacity, ultimately facilitating cancer progression [54]. Clinically, many OKC lesions are accompanied by infections and inflammatory cystic fluid, highlighting the need for further investigation into whether carcinoma in OKC is associated with a pronounced inflammatory microenvironment.

The phenotypic transition of fibroblasts is considered pivotal in the malignant transformation of OKC, particularly the shift from IFBs to MFBs. Previous studies have shown that the presence of myofibroblasts within cancer-associated fibroblasts can promote tumor development in colitis-associated tumors and increase the proportion of cancer stem cells in early colorectal cancer [55], [56]. Similarly, in ovarian cancer, adipose-derived mesenchymal stem cells differentiate into myofibroblasts via the TGF-β1 signaling pathway, contributing to the establishment of the metastatic microenvironment by activating MMP2, MMP9, and promoting EMT to enhance cancer proliferation and dissemination [57]. In the context of oral submucous fibrosis (OSMF), the emergence of myofibroblasts is considered a sign of exacerbated fibrosis, potentially altering the OSMF microenvironment and promoting cancer development [58]. At the protein level, S100A6 is recognized as a universal marker of activated myofibroblasts, consistent with our observations in the COKC microenvironment [59].

Clinical data analysis of 13 COKC patients revealed that 11 cases (84.6 %) reported pain at the primary site, with more than half experiencing radiating neuropathic pain. A comparison with clinical data from 277 OKC patients identified pain as the most prominent clinical feature of COKC. A literature review on odontogenic keratocyst malignancy found that 78.95 % of patients experienced varying degrees of pain, consistent with our findings [6]. These results suggest that unexplained persistent preoperative pain could serve as a potential indicator of OKC progression to COKC.

Through single-cell data, typical images of COKC intracranial invasion, and IHC staining, we hypothesize that COKC may exhibit neuroinvasive potential. During tumor progression, nerve-induced sprouting and infiltration into the tumor can promote interactions between tumor cells and neurons, facilitating tumor spread—a process termed perineural invasion (PNI). PNI is widely observed in various malignant tumors, including cervical, bladder, prostate, gastric, head and neck, and pancreatic cancers [60]. In head and neck cancers with intracranial spread, nerve pathway invasion is often linked to poor prognosis and severe pain [61]. In the COKC samples we analyzed, the NRG1/ERBB4 receptor-ligand pair mediated the strongest interaction between neuronal and tumor cells. Pancreatic cancer studies further support our findings, showing that NRG1 promotes neuroinvasion by activating the ERBB2/4 receptor-mediated autocrine loop [62]. The high expression of NRG1 receptors in the C0 subpopulation suggests that, unlike the C3 subpopulation, which represents the final stage of malignant differentiation, the C0 subpopulation may exhibit stronger neuroinvasive capacity despite its lower stemness characteristics. Therefore, the C0 subpopulation could act as a potential biomarker for predicting neuroinvasion in COKC patients.

Due to the neuroinvasive potential of COKC, particularly when the inferior alveolar nerve in the mandible is affected, precise intraoperative mapping of the nerve is essential to ensure the complete resection of the affected nerve tissue. In these cases, tumor resection alone may not be sufficient for complete clearance and must be accompanied by meticulous nerve management to prevent residual lesions. For cases with extensive nerve invasion, complete excision of the affected nerve is crucial to ensure surgical success and minimize the risk of recurrence.

Furthermore, our clinical data indicate that COKC generally exhibits low lymph node metastatic potential. Using single-cell sequencing, we have made preliminary inferences about the biological mechanisms underlying this phenomenon. In addition to T cell activation, reduced EMT activity and reduced VEGFC expression, MAST4 may also play a role in the low cervical lymph node metastatic potential of COKC. Previous studies have associated MAST4 mutations with neurodevelopmental disorders, developmental delays, and infantile spasms [63]. However, reports linking MAST4 to cancer are extremely rare, and no studies have explored its role in head and neck tumors. Our hypothesis requires further validation through functional experiments.

Previous studies have frequently recommended ipsilateral radical or functional cervical lymph node dissection for COKC [6]. However, given the low lymph node metastatic potential of COKC, we suggest that biopsy of enlarged lymph nodes or selective cervical lymph node dissection may be a more appropriate approach.

While providing valuable insights, our study has several notable limitations. Firstly, the rarity of COKC limited the availability of fresh samples for single-cell sequencing, underscoring the need to validate our findings in a larger patient cohort. Secondly, the retrospective nature of our clinical data, involving 13 COKC patients from a single medical center, introduces the risk of selection bias due to the lack of a validation cohort. We advocate for future large-scale, multicenter studies to validate our biological findings more comprehensively. Thirdly, the absence of cell lines, organoid models, and in vivo tumor models for OKC and COKC hampers direct validation of key molecular targets’ functions and mechanisms.

In summary, our study provides a comprehensive cellular map of COKC tumor cells and their microenvironment, highlighting their transcriptional features, malignant differentiation pathways, and fibroblast characteristics. Additionally, we detailed the clinical features of COKC, offering valuable insights into the mechanisms underlying OKC transformation into malignant tumors.

## Ethics approval and Consent to participate

The study protocol was approved by the Ethics Committee of West China Hospital of Stomatology, Sichuan University (Document number: WCHSIRB-D-2022–213), and all research adhered to the Declaration of Helsinki principles.

## Funding

This study was supported by the 10.13039/501100001809National Natural Science Foundation of China (Grant number: 2023NSFSC0704) and Exploration and Research Project in West China Hospital of Stomatology, Sichuan University (Grant number: LCYJ2023-DL-1).

## CRediT authorship contribution statement

**Guanru Wang:** Writing – original draft, Data curation. **Grace Paka Lubamba:** Writing – original draft, Data curation. **Mingzhe Bao:** Writing – original draft, Data curation. **Hongling Li:** Visualization, Resources. **Chunjie Li:** Writing – review & editing, Methodology, Conceptualization. **Taiwen Li:** Formal analysis. **Yaling Tang:** Writing – review & editing, Investigation. **Bo Han:** Investigation, Data curation. **Yike Li:** Methodology, Formal analysis. **Guile Zhao:** Writing – original draft, Formal analysis, Data curation, Conceptualization.

## Declaration of Generative AI and AI-assisted technologies in the writing process

No artificial intelligence (AI) and AI-assisted technologies were used in the writing process.

## Declaration of Competing Interest

The authors have no conflicts of interest relevant to this article.
