# Supplementary material for: Integrating single-cell sequencing and clinical insights to explore malignant transformation in odontogenic keratocyst
Source: Comput Struct Biotechnol J. 2025 Mar 18;27:1158–72. doi: 10.1016/j.csbj.2025.03.027 (PMC11981763; doi:10.1016/j.csbj.2025.03.027)
Supplement: Supplementary file 1 — Supplementary material [file mmc1.docx]

**
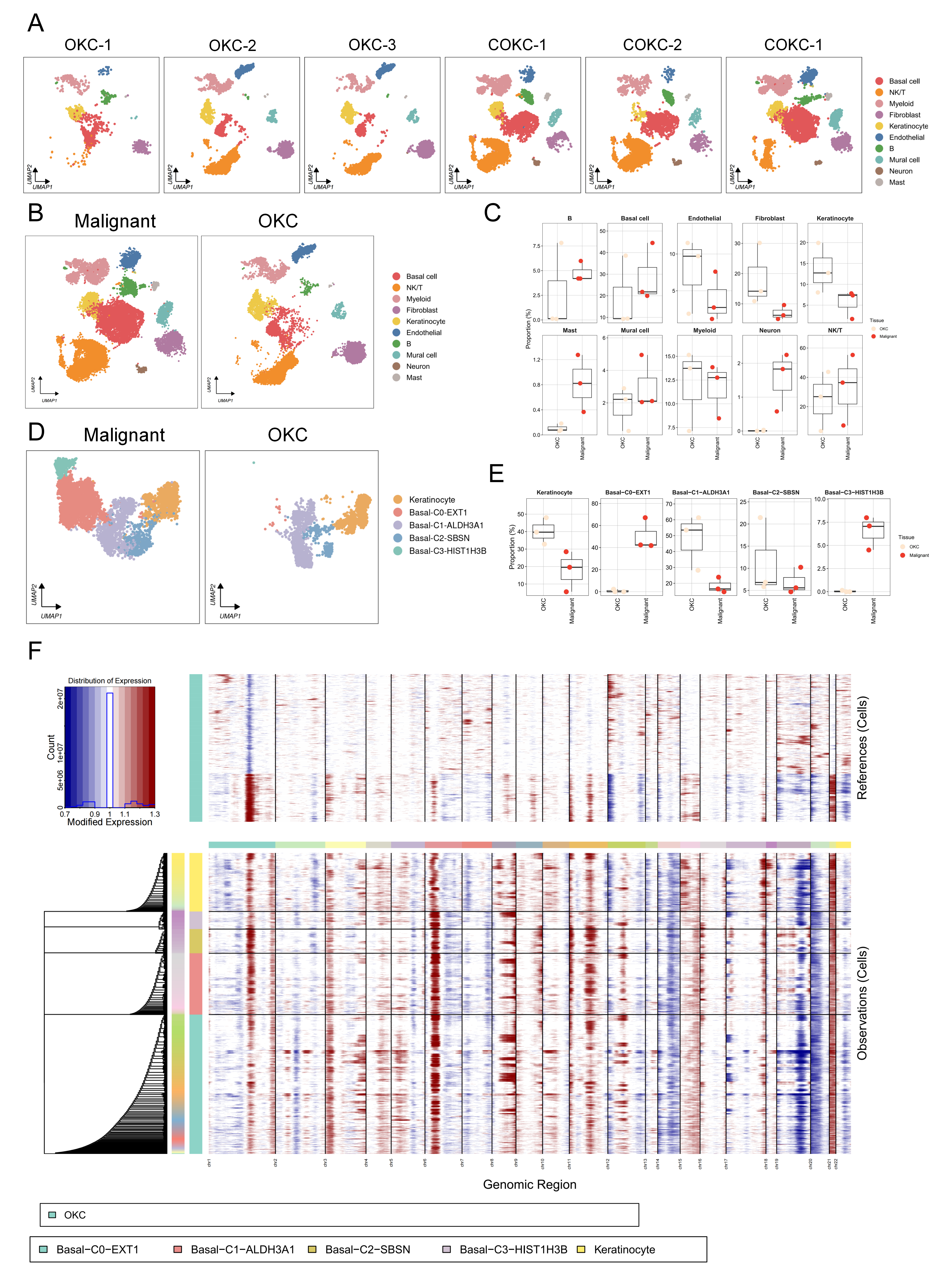
**

**Figure S1.** (A) UMAP plots representing various cell subpopulations across different samples. (B) UMAP plots representing various cell subpopulations across various tissue types. (C) Bar chart displaying the proportional differences of major subpopulations between OKC and COKC tissues. (D) UMAP plots showing the distribution of different epithelial subgroups across various tissue types. (E) Bar chart displaying the proportional differences of epithelial subpopulations between OKC and COKC tissues. (F) Heatmap of chromosome-wide CNV signals in single cells detected by scRNA-seq, using epithelial cells from OKC samples as a reference.

**
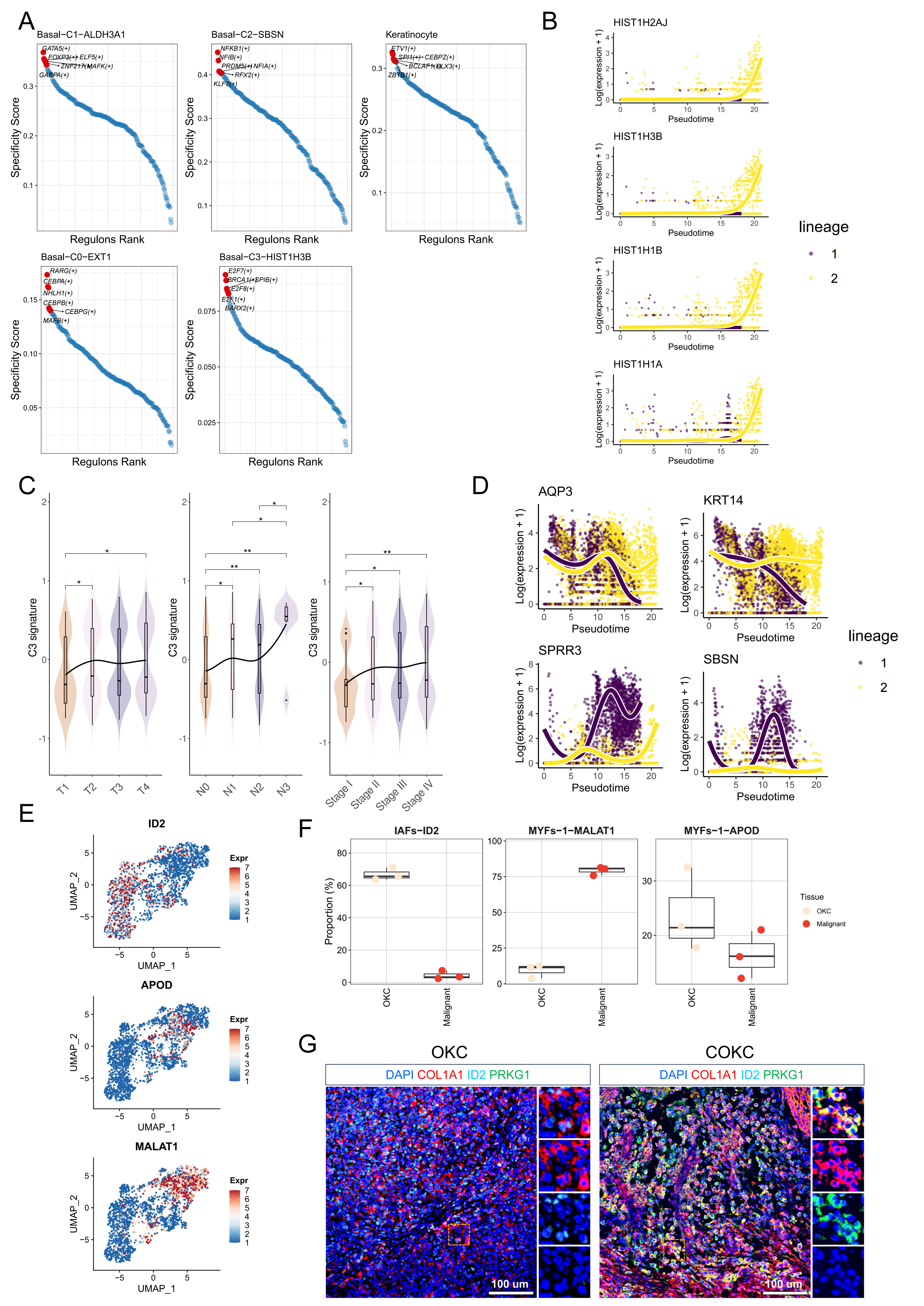
**

**Figure S2.** (A) Rank dot plot showing the six most active transcription factors in each epithelial subgroup. (B) Time course plot of histone-related gene expression in the C3 subgroup. (C) Expression levels of the C3 signature across tumor size, lymph node metastasis, and clinical stages in the TCGA-HNSC cohort. (D) Time course plot of the genes of interest (KRT14, AQP3, SBSN, SPRR3) over time. (E) FeaturePlots showing the distribution of fibroblast marker genes in the UMAP plot. (F) Bar chart displaying the proportional differences of fibroblast subpopulations between OKC and COKC tissues. (G) mIHC staining images showing the abundance differences of IFBs-ID2 and MFBs-MALAT1 between OKC and COKC.

**
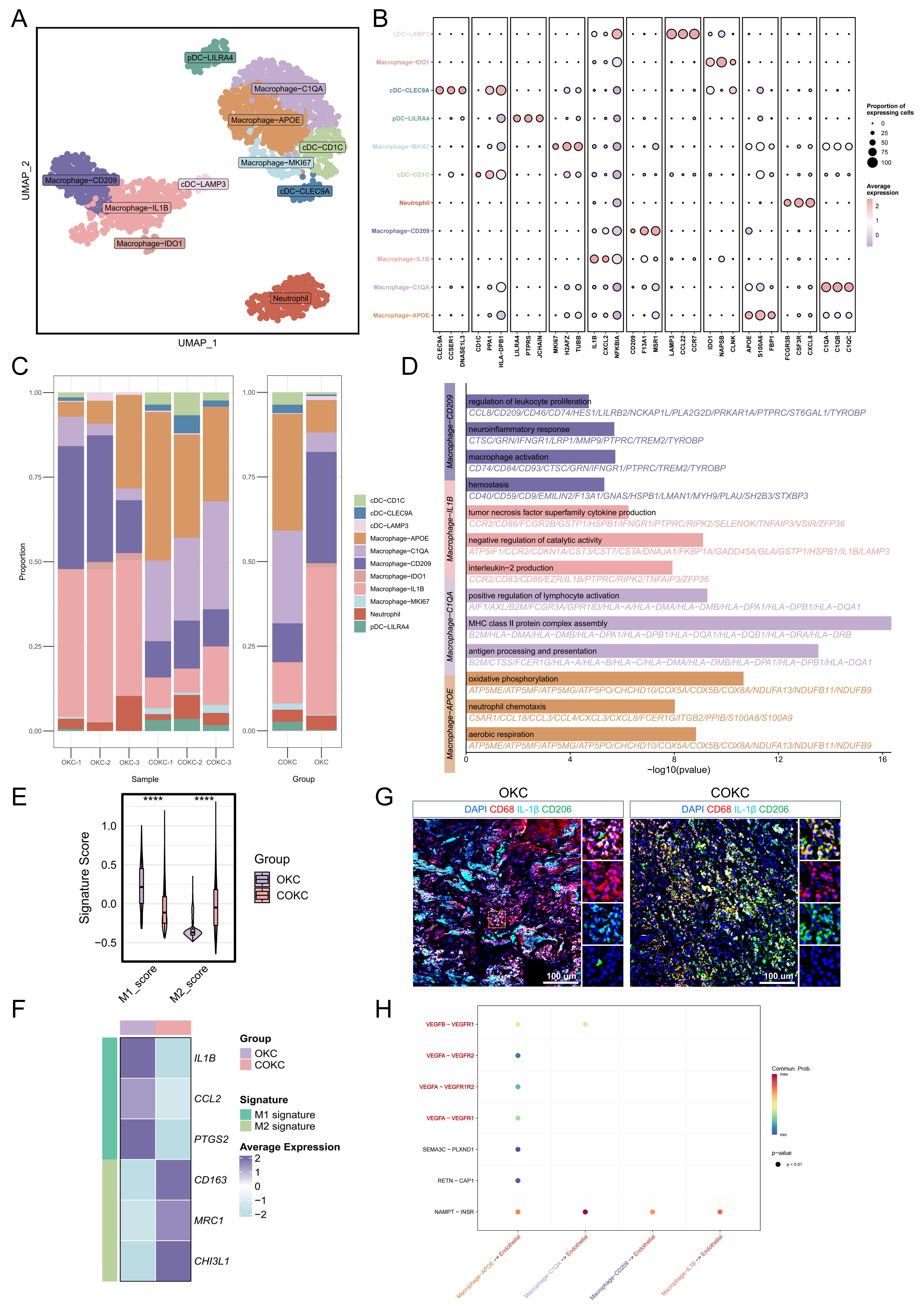
**

**Figure S3.** (A) UMAP visualization of myeloid cell subpopulations in OKC and COKC. (B) Dot plot showing the expression levels of key marker genes across different myeloid cell subpopulations. (C) Bar plot depicting the proportion of myeloid cell subpopulations across different samples (left) and grouped by OKC and COKC (right). (D) Gene ontology (GO) enrichment analysis of differentially expressed genes in myeloid subpopulations. Key biological processes associated with each macrophage subset are highlighted. (E) Violin plot comparing the M1 and M2 macrophage signature scores between OKC and COKC groups. (F) Heatmap of differentially expressed genes related to M1 and M2 macrophage polarization in OKC and COKC. (G) mIHC staining images showing the abundance differences of Macrophage-IL1B and Macrophage-CD206 between OKC and COKC. (H) Cell-cell communication analysis illustrating ligand-receptor interactions between macrophages and endothelial cells in COKC.

**
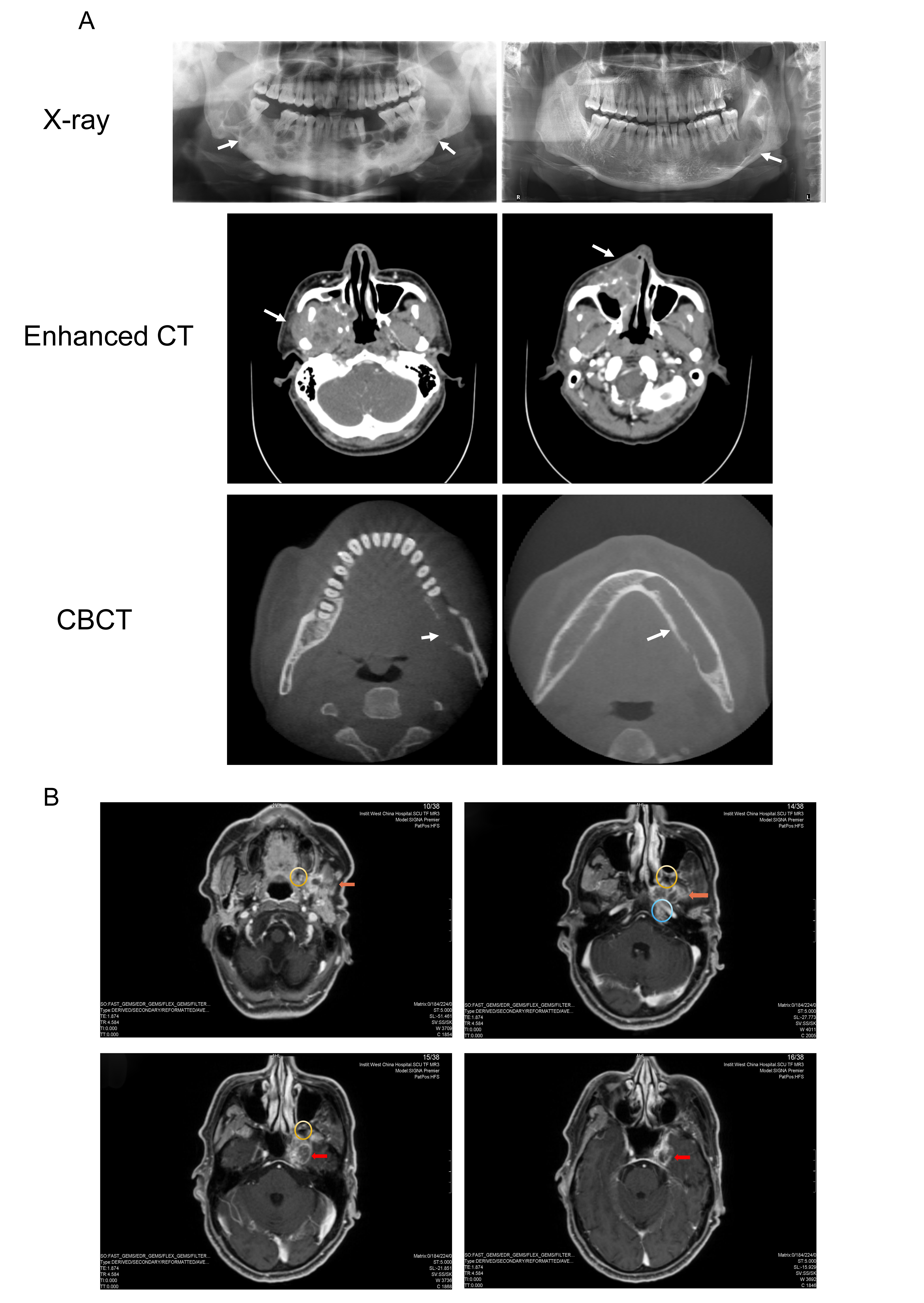
**

**Figure S4.** (A) **Radiological features of COKC patients under different imaging examinations.** Panoramic X-ray reveals multilocular (left) or unilocular (right) radiolucencies, with multilocular being a typical manifestation of Gorlin syndrome. Enhanced CT of the maxillofacial region reveals typical cystic-solid images within the lesion area. CBCT imaging demonstrates a distinct unilocular osteolytic lesion within the jawbone. (B) MRI images of a patient with recurrent COKC resulting in intracranial invasion (orange arrow: previous operation area and recurrence focus, red arrow: intracranial invasion focus, yellow circle: pterygopalatine fossa, blue circle: rupture foramen).

**
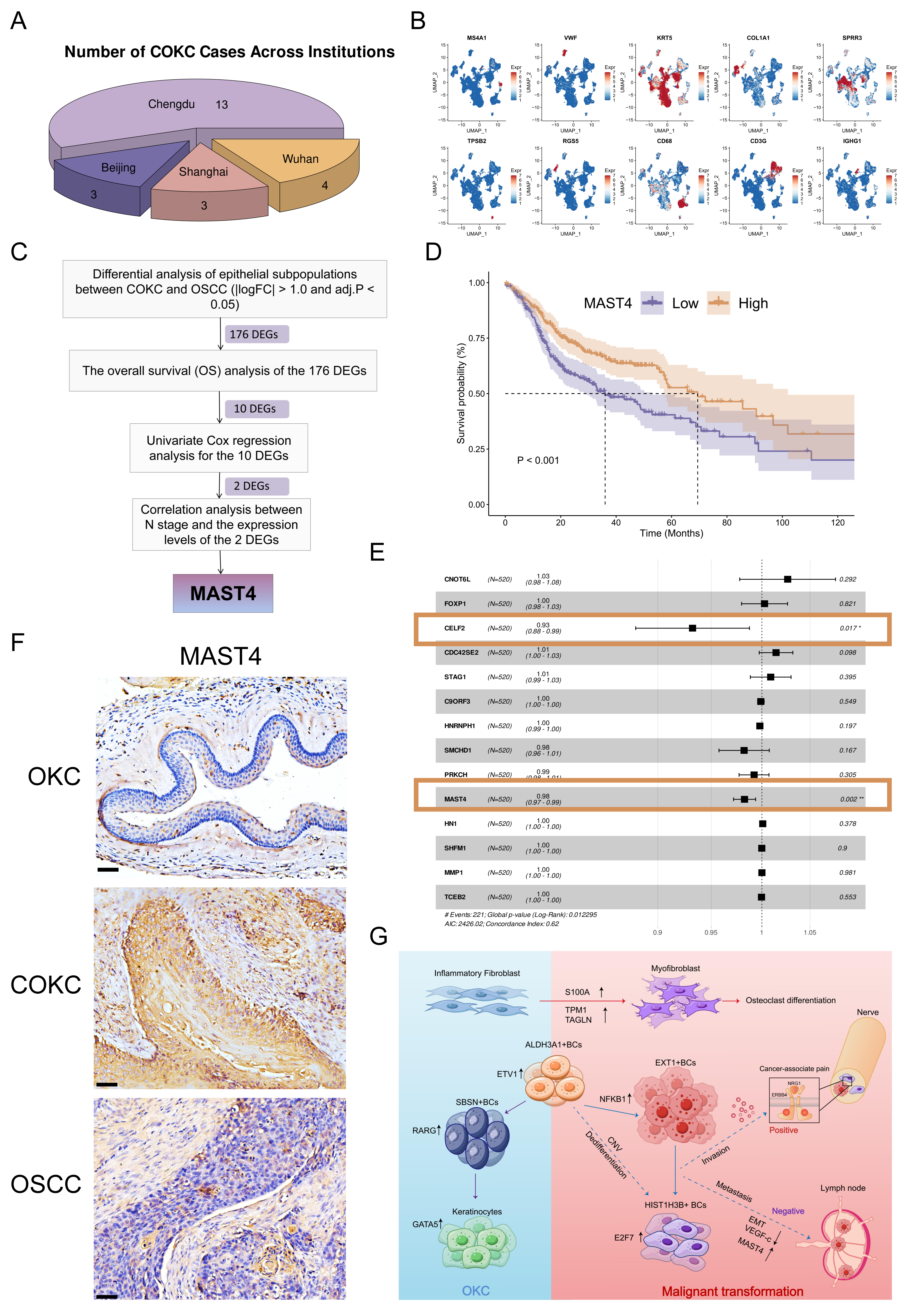
**

**Figure S5.** (A) Pie chart showing the number of COKC cases across four research institutions. (B) FeaturePlots of marker genes in the integrated atlas of COKC and OSCC. (C) Flowchart illustrating the identification of MAST4 through differential expression analysis, survival analysis, Cox regression, and correlation with N stage. (D) Kaplan-Meier survival analysis showing significant differences in overall survival between high and low MAST4 expression groups (*P* < 0.001). (E) Forest plot of univariate Cox regression analysis for DEGs, highlighting CELF2 and MAST4 as significant prognostic markers for overall survival in the TCGA-HNSC cohort. (F) Immunohistochemical staining of MAST4 in OKC, COKC, and OSCC tissues (scale bars, 50 µm). (G) The biological mechanism and clinical manifestations of malignant transformation of OKC discovered in this study were summarized.
